# Perspectives of eFootball Players and Staff Members Regarding the Effects of Esports on Health: A Qualitative Study

**DOI:** 10.1186/s40798-023-00617-0

**Published:** 2023-07-26

**Authors:** Ana Monteiro Pereira, Caroline Bolling, Phil Birch, Pedro Figueiredo, Evert Verhagen, João Brito

**Affiliations:** 1 Portugal Football School, Portuguese Football Federation, Oeiras, Portugal; 2 Research Center in Sports Sciences, Health, Sciences and Human Development, CIDESD, ISMAI, University of Maia, Maia, Portugal; 3 Amsterdam Collaboration on Health and Safety in Sports, Department of Public and Occupational Health, Amsterdam Movement Sciences, Amsterdam UMC, Vrije Universiteit Amsterdam, Amsterdam, The Netherlands; 4 Institute of Sport, Nursing and Allied Health, University of Chichester, Chichester, PO19 6PE UK; 5 Physical Education Department, College of Education, United Arab Emirates University, Al Ain, Abu Dhabi, UAE; 6 Research Center in Sports Sciences, Health, Sciences and Human Development, CIDESD, Vila Real, Portugal

**Keywords:** Competition, eFootball, FIFA, Healthcare, Lifestyle, Mental health, Physical health, Sports medicine, Well-being

## Abstract

**Background:**

Recently, esports have been argued to impact esports players' health, particularly for those competing at higher levels. Esports are a relatively new phenomenon, and an inside perspective regarding esports players’ needs and experiences is essential to promote adequate health support for this population. Thus, in this qualitative study, we explored the perspectives of elite esports players and staff members regarding the effects of esports participation on health. Ten semi-structured interviews were performed with members of the Portuguese FIFA (i.e. FIFA EA Sports^®^) eFootball National team (i.e. five elite electronic football players, one world-class electronic football player, two national team coaches, and two members of the esports department). Data analysis was undertaken following the principles of thematic analysis.

**Results:**

We identified four major superordinate themes: health definition (1), factors of esports that impact mental (2) and mental health (3), and strategies to improve esports players’ health (4). Esports-related factors such as gaming, competition, and performance were said to impact mental health, while equipment, facilities, and esports-related sitting time affect physical health. To minimise those risks, four main strategies were suggested: optimising and scheduling esports training, improving lifestyle habits with an emphasis on physical health, enhancing facilities and equipment, and improving health support, particularly with a mental health professional.

**Conclusion:**

Esports players and staff members are concerned and aware of esports’ mental and physical demands. Understanding what esports players need and perceive about their health, complemented with the view of staff members, and their proposed strategies for health promotion might help define and prioritise healthcare needs, which could help players and the broad esports community.

**Supplementary Information:**

The online version contains supplementary material available at 10.1186/s40798-023-00617-0.

## Key Points

1. Esports have been seen to affect esports players' mental and physical health.
2. Esports players may benefit from playing esports by improving cognitive ability and by its positive effects on mental health when players accomplish good performances.
3. Esports participation may be associated with symptoms of common health problems related to esports competition and low performance, while impacting physical health due to using equipment for long periods or in an incorrect way, besides the associated sitting time.
4. The main strategies suggested to improve esports players' health were optimising esports training and scheduling, improving lifestyle habits, enhancing facilities and equipment, and improving health support.

**Supplementary Information:**

The online version contains supplementary material available at 10.1186/s40798-023-00617-0.

## Background

The popularity of esports worldwide is noteworthy [1]. Currently, esports comprise regulated competitive video games [2]. The industry dynamics have shifted towards traditional sports models (e.g. official leagues, stable fan bases, and more extensive and consistent investments) [2], with several esports modalities now being part of competitions held by the International Olympic Committee (IOC) [3]. Likewise, as professional athletes, esports players need intense training and commitment to remain competitive while developing and mastering specialised skills [2, 4–7]. In fact, gaming has been positively associated with improved cognitive benefits such as perceptual-cognitive abilities, domain-specific skills, and a wide range of visuospatial and attentional tasks (e.g. psychomotor and cognitive speed, creativity, strategic thinking, problem-solving, anticipation, response mapping, attention, working memory) [8–10]. Nevertheless, aligned with the increased pressure to perform (e.g. pool prizes, media impact), professionalisation, and training and competition settings (e.g. dark rooms, play mechanics), theoretically, esports players might be prone to a unique set of health problems and injuries that would require proper healthcare attention [11–13].

Health at its fullest is the highest attainable standard for every human [14]. In 1948, the World Health Organisation (WHO) defined health as “a state of complete physical, mental and social well-being and not merely the absence of disease or infirmity”. Many perceive health as not being a dichotomous variable (i.e. healthy vs unhealthy) but one that occurs along a continuum between more healthy and less healthy [15], with more realistic views considering that people have the ability to adapt and to self-manage [16].

Recently, different problems affecting the health of esports players have been described, including mental health [6, 17, 18] and musculoskeletal problems [19–21], most of them being preventable with adequate and prompt care. Factors related to esports increased proficiency and competitive status [10, 17, 22], including perceived stress responses (e.g. nervousness and excitement before a competition and post-competition responses) [23] and the dependence on performance achievements [18, 24–26], have been reported to contribute to mental health symptoms in esports players [27] that could predispose to unhealthy lifestyles (e.g. caffeine or alcohol use, smoking habits) and other mental health problems (e.g. sleep disturbance) [17, 23]. Conversely, musculoskeletal problems (e.g. wrist or hand pain, back pain) have been reported in different studies on esports players [28, 29], and the risks associated with musculoskeletal problems may increase with excessive gaming [29]. Additionally, esports participation is sedentary, with players gaming between 3 and 10 h per day [30].

Hence, to provide adequate healthcare and protect esports players’ health, it is crucial to listen to the players and staff members involved in esports to understand their beliefs, attitudes, and needs regarding their health and the healthcare support currently given to this population. For that, qualitative research is an adequate approach to generate in-depth insights into beliefs, assumptions, values, or practices and is a valuable tool [31, 32]. Recently, qualitative studies have emerged in esports-related research, namely studies with players from different countries and esports modalities on subjective experiences of stressors and coping [22, 23, 33], perceptions of success determinants [24], perceptions of training effectiveness [34], and indexing esports performance [35]. Still, those problems and perceptions studied might depend on the context (e.g. esports modalities, country, esports settling development) [30, 36]. Moreover, besides esports players, team staff, particularly esports coaches, might give an essential insight into esports players’ health [37]. They frequently work as intermediaries between teams and players and can be the first contact of esports players when any health problem occurs. Yet, they are not usually involved in studies related to esports. Thus, unlike previous research, this study will include participants and staff members partaking in electronic football (eFootball) competitions, a specific esports modality. If players can give their insights on what they are already experiencing, staff members could help define what they can offer. Recently, entities like the IOC [3] have included sports-related esports in their competitions [38], so clarification on the perceived needs and current state of healthcare provision from players and staff members (providers) in this specific context might be necessary. Besides, knowing the heterogeneous development in esports teams and supporting structures worldwide, new insights on the perceptions of the current needs of esports participants will contribute to better preparing the structure of healthcare support in esports settings. Including staff providers (i.e. coaches and members of the esports department) will increase diversity of perceptions in the current status of health perceived by esports primary stakeholders. Additionally, those perceptions might differ between players and staff members, including esports coaches [39, 40].

Ultimately, the present study aimed to gain new insights and to understand the perceived needs regarding health and strategies to support esports players, to ultimately help promote adequate care for these participants, advancing the understanding of how esports players from higher competitive levels and staff members working with them perceive about health, the impact of esports participation and the health-care support currently given in esports circles. To do so, we qualitatively investigated the perceptions that esports players and staff have on health, how esports affect their health and potential strategies to improve esports players’ health in the esports context. Doing so, we hope to advance on previous literature that quantitatively and qualitatively has evaluated health-related parameters in esports players, which calls for more research in this area.

## Methods

### Ontological and Epistemological Assumptions

This qualitative study follows a critical realist perspective approach, which posits that we interact with an actual external world and that this world existed before our language, ideas, and concepts about it [41, 42]. Hence, knowledge of the world is deemed relative to historical context, our perspectives and interests, and the influence of others on us. As a result, any accounts of our understanding of reality are fallible [43]. With this epistemological and ontological position, a qualitative interview-based methodology was chosen to explore participants’ realities, experiences, and perceptions of what may cause such experiences [44].

### Participants

We recruited elite and world-class (adapting [45]) FIFA esports players (i.e. esports players that have at least one call to be part of the Portuguese eFootball national team and with competition-relevant tricks and performance achievements or being in the top three at a major international event such as the World Championship, respectively), the Portuguese national team coaches, and the coordinators of the Portuguese Football Federation (FPF) eFootball department for this study. With this sample, we included primary stakeholders [38] comprising the eFootball national team. Only participants older than 18 years were included.

### Procedures

The present study was conducted in collaboration with the FPF and the FPF electronic Football (eFootball) department, responsible for the coordination of the National eFootball team training camps and the National eFootball League (e.g. FPF Digital Challenge, FPF Portuguese Cup, etc.). Aiming to capture a wide range of perceptions of primary stakeholders in the esports ecosystem [38], purposive sampling was used to identify and select the sample group.

Following the lead author’s institutional data protection policy and explaining the study's main goals, the FPF eFootball department provided a list of potential participants for the study. Those who expressed interest in participating gave their contact details to directly arrange the interview with the principal researcher (AMP). Additionally, participants were asked to provide the contact of other further potential participants [46].

### Data Collection

Based on the provided contacts of potential participants given by the FPF eFootball department, 14 players that participated recently in the Portuguese eFootball national team and all four members of the staff were contacted. Six players and all staff members accepted the invitation, while eight players did not respond after three contact attempts. Yet, the last two players interviewed and included in the present study did not add new information, indicating data saturation [47]. All participants provided oral informed consent before the interview. After the interview, the participants were asked to complete an anonymous online form using SurveyMonkey^®^ for sample characterisation. This survey included questions related to age, gender, education, occupation, and sports registration status (i.e. registered in a sports federation in any other sports) and esports participation. The study was approved by the Portugal Football School Ethics Committee (CE PFS 01.2021).

### Interview Guide

Individual semi-structured interviews were held and recorded online using Microsoft Teams^®^ by the first author (AMP) between January 2022 and April 2022. The interviews were scheduled depending on the participants' availability. The interviews followed two flexible interview guides, one for esports players and one for staff members (see Additional file 1) that slightly differed depending on participants' activity (e.g. players: “*Did you already feel any health problems due to your esports practice?”* versus staff: “*what health problems are more frequent in esports players?*”; players: “*What type of healthcare services are usually given by esports teams?*”; staff: “*What kind of medical support do you think you could/should have for your elite esports practice?*”). This interview format allowed the interview guide to be supplemented by follow-up and probing questions according to participants' responses, allowing us to explore new topics raised by participants [48]. AMP developed the interview guides comprising three main topics: (1) health definition, (2) health effects (positive and negative) of esports participation, and (3) healthcare for esports participants. The interview guide was discussed with CB, PB, and JB to enhance rigour (e.g. discussion flow, starting with broad questions to more specific ones). CB and PB are familiar with qualitative research, having developed qualitative studies in sports and esports contexts. Indeed, a pilot interview was conducted with a Portuguese recreational gamer working as a community manager for elite esports players and by a researcher familiar with using qualitative methods; this led to minor changes in the initial questions (e.g. “*affects your health*” changed to “*players’ health*”) and was crucial to avoiding jargon that could be used by AMP as a healthcare professional. Indeed, the first two interviews were reviewed by CB during a meeting to evaluate how AMP conducted the interviews.

Other follow-up questions were used during the interviews to clarify any points (i.e. “*How do you think esports practice affects esports’ player health? And here it can be positively or negatively”*; “*when you talk about “it’s mental”, what do you mean by that?”).* A last question was added at the end of each interview, asking for feedback on the interview and describing aspects the participants thought were important but were not part of the questions (i.e. “*If I wanted to know more about the health of esports players and their perspectives on health, what should I have asked or what could we have talked about more during this interview?*”). All interviews were conducted in Portuguese, the native language of all participants and the first author.

### Data Analysis

Data collection, analysis, and manuscript redaction considered Tracy’s “big tent” criteria to enhance rigour [49]. The interviews were video recorded, transcribed verbatim (AMP), and then repeatedly read and analysed by two researchers (AMP and CB; step 1).

Pseudonyms were randomly selected using typical Portuguese names (aligning to the participants' gender) and replaced participant names to assure anonymity in all data collection. Chosen names consider the initial letter of each name according to participants’ activity (i.e. J = Player, C = Coach, D = esports Department). Collected data from the 10 interviews were analysed following the six steps of thematic analysis proposed by Braun and Clark [50]: (1) familiarisation with data, (2) generation of initial codes, (3) search for the more mentioned themes, (4) review themes based on frequency, (5) define and name themes primarily according to frequency, then based on patterns of meaning and shared core concept, and (6) produce the report with process description. Some steps were frequently overlapped for a more rigorous data analysis (e.g. steps 2 and 3, 5, and 6). Thus, the final superordinate, subordinate, and ordinate themes were data-driven, following a stepwise process of coding and re-coding with an inductive approach to thematic analysis [51, 52]. Therefore the final themes do not reflect the questions asked during the interview nor the research team’s beliefs [50, 51].

All data analysis was done in Portuguese (the native language of AMP, CB, and JB). The selected quotes were translated to English by AMP and verified by JB and an official translator. CB guided all stages; the researcher has vast experience with qualitative research, including thematic analysis. Given the volume and complexity of the data collected, data were reanalysed several times, based on the input of CB, for a more concise data presentation, ultimately to respond to the study goals. JB, who was an interviewed-blind author, participated in the last 3 steps to sense-check the preliminary themes and report, contributing with their sports and exercise background and analytic thinking. Any disagreement with coding was settled through discussion between AMP, CB, and JB during five online meetings throughout the process. Initial disagreements regarding data presentation were resolved for a more concise data presentation with three main topics. Self-reflection, aligned with mentoring and supervision from CB, PB (who also has a vast experience with qualitative research), and JB, was crucial to guarantee “transparency”, thus minimising any personal bias and preconceptions the first author could have due to their experience in clinical practice. The present qualitative study follow the Consolidated Criteria for Reporting Qualitative Research [53] (Additional file 2).

## Results

In the present study, the most debated themes were grouped into four superordinate themes: the participants’ definition of health, factors of esports that impact health (mental health and physical health), and strategies used to improve esports players' health in general, as shown in the following sections (Table 1 and Additional file 3: Fig. S1).

**Table 1:** Superordinate themes, subordinate themes, and themes

Superordinate theme	Subordinate theme	Themes
Health definition	General health definition	General health definition
	Mental health definition	Mental health definition
	Physical health definition	Physical health definition
Factors affecting mental health	Gaming	Cognitive ability
	Competition	Symptoms of mental health problems
		Addiction to competition
		Mental fatigue
	Performance	Sense of achievement
		Symptoms of mental health problems
		Performance anxiety
		Excessive gaming
Factors affecting physical health	Equipment and facilities	Eye problems
		Poor postures
		Musculoskeletal problems
	Sitting time	Sedentarism
Strategies to improve esports players’ health	Optimising esports training and scheduling	Time management
		Scheduling daily routines
		Having other activities
	Improving lifestyles	Physical activity
		Other lifestyle habits
	Enhancing facilities and equipment	Examples of improvements to facilities and equipment
	Improving health support	Health prevention
		Mental health support
		Other examples of health support
		Health-related research

Participants answered without distinguishing eFootball or esports in general.

Gaming, performance, and competition related to esports were the most significant subordinate themes illustrating factors affecting players’ mental health. At the same time, equipment, facilities, and esports-related sitting time were the main factors affecting esports players’ physical health. For all those factors, different strategies grouped into four main subordinate themes were suggested to minimise esports-related physical and mental health consequences. The mean duration of the interviews was 37 ± 10 min (range: 28–55 min; see Additional file 1).

### Sample Characterisation

Ten participants were included in the study: six male eFootball players partaking in FIFA eFootball competitions (*M* = 22 ± 3, ranging from 18 to 26 years of age) and four male members of the staff, which included the coaches of the eFootball national team and coordinators of the FPF esports department (*M* = 32 ± 3, ranging from 28 to 35 years of age). Three out of four members of the staff were former esports players.

All interviewed players profited financially from esports and were part of an esports team; yet, only three were exclusive esports players. The players usually practised esports between 1 and 8 h per day (*M*_*h.min*_ = 4.15 ± 2.29 per day) for 3–7 days a week. Recreationally, four players also spent 1–3 h per week playing other video games (*M*_*h.min*_ = 1.30 ± 1.23 per week). Additionally, all staff members were also recreational gamers, spending 2.00 to 10.00 h per day gaming. Nine participants usually practised recreational sports besides esports. In the year data collection occurred, the Portuguese eFootball National team ranked first on the FIFA eNations ranking and won the 2022 FIFA eNations Cup.

### Health Definition

#### General Health Definition

In line with the WHO definition of health, well-being was frequently subdivided into physical, mental, and social well-being, along with being healthy, feeling well, and “having no diseases”, were the subordinate themes used to define health, as explained by *Joaquim: “ends up as being … about physical, mental, social well-being and more than all of this, perhaps we aren’t only well in these states, but we feel well in ourselves. I think that’s the main thing”.*

In fact, they struggle to give their own words to the definition.

*José: “Health might be… It’s difficult to explain what it is, but I would say that maybe something that make us, I mean, something that we think to make us feel good on a daily basis and being healthy means being ok, not being sick and, obviously, do not have any worries about us… I think it’s hard to explain without using the word.”*

Besides, all participants noted that "health" encompassed both physical and mental health, with eight participants stating that good mental health is needed for good physical health and vice versa. This is exemplified by Duarte that defined health as *“if a person is well in their health, has no disease, has anything similar associated …there are various types of health considered: physical, mental health.”*

#### Mental Health Definition

As exemplified by Duarte that stated that mental health is *“not having any type of problems, whether depressions, whether stresses, … it’s about being aware of everything that is going on.”,* “Mental health" was perceived as being happy and not having any symptoms of mental health problems, such as depression, stress, or anxiety, and essential for players’ well-being.

In line with Jonas that said “Mental health is *psychological well-being, being secure in yourself, confident”,* the other two players added that mental health encompasses the feeling of confidence, motivation, and well-being.

#### Physical Health Definition

“Physical health” was described as being physically active, with no mobility problems, and independent in daily living activities, as exemplified by Duarte: *“the physical is the motor level and everything else […] doesn’t depend on anything, nor on anybody for doing the basic and daily things.”*. Conversely, physical health was also associated with having no health problems or diseases.

*Joel: “that’s the health people talk about when we …hen we are sick or when we have some health problems … […] for example obesity or sometimes a more serious type of disease, cancer and those things.”*

### Factors of Esports that Impact Mental Health

The (subordinate) themes identified by the authors following data analysis as impacting mental health were “gaming”, “competition”, and “performance”. In this context, gaming refers to playing video games, which is needed for recreational and esports players; competition refers to all organised competitive events where esports players participate; and performance is gauged by the results obtained in esports competitions [54]. Esports was considered a mentally demanding activity, where focus, quick thinking, and good mental health are key to motivation and success in esports performance.

#### Gaming

Gaming was argued to positively impact cognitive ability by helping players work and develop reflexes, thinking capacity, quickness of thought, multitasking ability, game perception, and knowledge:

*Carlos: “the brain gets a lot quicker, for example, you become able to develop various … various tasks at the same time, what they call multitasking, … your brain becomes more flexible, […] the cognitive capacities always ended getting better worked … and explored … and increased.”*

Besides improving other daily activities, those factors were also considered needed to reach the highest competitive scene.

*“David: [players] can benefit very much from this competitive practice, above all by really stimulating the brain, […] the question of reflexes and also the actual knowledge that you have to have.”*

#### Competition

Different participants suggested that esports competition could predispose or exacerbate pre-existing symptoms of mental health problems (i.e. anxiety and sleep problems) and promote addiction (i.e. to competition and winning at all costs) and mental fatigue.

*Joaquim: “because at this level, the competition is not going to be physical, it’s mostly going to be mental, it’ll cause us a lot of … a lot of emotions, much anxiety, much stress, much frustration …. real exhaustion.”*

David also added that: *“Players can feel a bit addicted to the competition, to the playing of the game and not knowing when to stop, how to stop, how to take a break.”*

Moreover, esports competitions were considered a "strenuous mental activity (Carlos)" where players feel exhausted after competition moments, even when competing for relatively short periods, as exemplified by José: “ *the playing of esports always leaves me very tired […], many hours and when we have to be very concentrated and very focused, and this ends up leaving us with this feeling of tiredness despite not having done any actual sport”.*

#### Performance

All participants acknowledged the mental health impact of performance, which is weighted on competition results. Therefore, when players accomplished their performance goals, they improved their sense of achievement, felt happier, and felt more confident with better self-esteem, which was repeatedly stated to improve mental health, as noted by Júlio: “*when things are going well, when the season is going well, is a good one, when all the work is going well, it very positively affects your mental health”.*

Conversely, bad results were explained to unveil or promote mental health problems, frequently described as “sadness”, “frustration”, “despair”, “isolation”, and even “depression” (e.g. *José: whatever the performance that you want to have, may even end up leaving us sad, anguished, frustrated*), performance anxiety (e.g. *David: These problems of anxiety, of stress… happen a lot. And they even happen in music and in other areas where there is a lot of social pressure, the spectacle … the performance. […] the other side of the coin is a bit, after this accumulated pressure, that then becomes a pressure cooker and can … and can really fall apart)* and excessive gaming (e.g. *Jaime: Players who are full-time FIFA, have FIFA as their focus and very often can exaggerate the number of hours they put into the game. It may even be unnecessary at times*).

According to all interviews, common mental health symptoms and other personal stressors (e.g. being a professional player) could hinder performance by interfering with cognitive ability and attention to gameplay, ultimately creating a vicious circle of thinking and behaviour (i.e. decreased performance leading to mental health symptoms and vice versa). Carlos explained that as “*If we have expectations, that are just too high and then we are not able to meet them or similar…the biggest and most various reasons can … can bring this, this kind of anxiety […] that turns into a snowball, doesn’t it?. If the results are going to bring … more or less pressure in accordance with what we can do.”.*

### Factors of Esports that Impact Physical Health

The other major theme derived from the interviews was physical health. All participants associated esports with physical health problems, primarily due to equipment and facilities used while playing and related sitting time.

#### Equipment and Facilities

Gaming equipment (i.e. monitor, gaming chair and controller) and the facilities where competition and training generally occur (i.e. indoors and dark environments, artificial lights) were discussed to affect physical health in the short (e.g. *Joaquim: I already feel some pain or other, when it’s an exhausting day of competition … I get to the end and already feel something”)* and long term (e.g. *Carlos: In the long term … I speak for myself … I feel that some bad habits of playing in front of television […]., can help with deteriorating and bring some diseases, such as myopia).*

Accordingly, the main problems associated with equipment were eye problems, poor postures, and musculoskeletal problems, while the facilities were mainly responsible for eye problems. Indeed, five players and one coach mentioned that they had already suffered from vision problems, dry eyes and headaches while playing esports, which tended to compromise performance.

*Jaime: “When I spend a lot of time in front of the monitor, I feel some tiredness, headaches, a slight discomfort and also a bit in my eyes.”*

Both players and staff members discussed that esports players are likely to play in what they described as incorrect postures (e.g. close to the monitor or reclined on the chair) that were highlighted to contribute to back pain and poor body posture. Two players reported they had already felt discomfort and stiffness while playing.

*César: “Having a bad posture, we’re always going to try and conform with it and if we get used to playing the game like that, it’s not going to have major implications if we always play in that position, if we always compete like that. The problems are later, in the long term.”*

Repeated movements and inappropriate gaming equipment, mostly in stiff positions, contributed to musculoskeletal problems, comprising local pain or minor injuries on fingers, hands, back (especially lumbar region), and knees, which generally did not require medical intervention.

*Júlio: “In terms of future health problems, it’s the … for me, it’s the … knees and knee and posture problems. […] If your posture is not correct, …, backpains […] problems with your bones, in your fingers due to being always making movements and I don’t know … the tension.”*

#### Sitting Time

*César: “The fact of … there being a lot of correlation with videogames being … being kind of sedentary … related to sedentarism. I think that they can bring some of the complications associated with this.”*

Increased sitting time related to esports practice was discussed to impact overall health, impairing eating habits and contributing to musculoskeletal problems while also exacerbating existing problems due to prolonged use of the equipment and incorrect postures for long periods.

*José: “We do not unwind, we spend a lot of time sat down. Obviously, we do not burn the calories that we need to take on and this is going to affect our physical condition negatively.”*

### Strategies to Improve Esports Players’ Health

Four main strategies were suggested to improve esports players' health.

#### Optimising Esports Training and Scheduling

*Joel: “There’s a lot of that idea of “the more hours you put in, the better you’re going to be”, but not always. Perhaps four hours well worked are worth more than eight hours not well worked.”*

Having appropriate time management (e.g. more efficient training sessions combined with physical activity, rather than spending long hours practising esports), scheduling pauses between games and training sessions and including periods for other activities were reported as strategies for minimising mental and physical health risks and increasing performance. This has been mostly explained by the member of the esports department:

*David: “A well-defined timetable, with a well-defined rhythm, almost a well-defined routine so that they are always the best prepared, not only at the level of the state of spirit but also at the physical level, being prepared for the activities.”;*

*Duarte: “They should have, to safeguard against the micro-cycles, more breaks. […] That is, the player is sat down for twenty minutes, there should afterwards be a break of five to ten minutes in compensation. […] A player should pay as much attention to the moments of rest as to the moments of competition.”*

For that, as exemplified by Jonas: *“I switch off completely from video games. I go to the cinema and see a film, I try not to …. I try to really rest my working instrument that is my hands … the players that turn in the best performances are normally the people that are able to distract themselves from this world and have a social life and an active physical life […] I always try and keep myself occupied with various things so that my thoughts are not only focused on this because I think it’s important to have this outlet, this means of escape”* having other activities (e.g. studying, being with friends, having other activities and life goals, playing sports) were examples of activities to refrain from esports, thus optimising esports training. Moreover, to prevent musculoskeletal problems, participants explained that they usually decide to have other activities with no screen or sitting time involved.

Those strategies were also said to prevent mental health problems related to low performance.

*José: “Doing some of the activities that can help me build my self-esteem, good thoughts, relaxation, distracting myself, thinking about various things. […] That’s it, playing sport or going out for a walk […] even when I lose, I go out to train [physically] because whenever I arrive home after this training, I feel very good about myself.”*

#### Improving Lifestyle

*Jaime: “Also looking after ourselves, that is, to play some sport. I think that’s also health. Having a balance diet, having time to sleep, at least eight hours or about that, at least in my case.”*

Avoid drinking or smoking, having (or aiming to have) a careful diet, improving sleeping time, and being physically active were lifestyle habits mentioned to improve esports players’ health.

*Carlos: “Those behaviours that we say are healthy or that science believes are correct, to remain capable. […] Avoiding those, avoiding those less good habits both in terms of diet and in terms of sleeping, even smoking and drinking.”*

Besides, all participants indicated physical activity as one of the most common habits to promote health, noting the positive impact of physical activity on mental and physical health.

*Carlos: “I think that physical activity helps ...… us to, to unwind. I don’t know what exact word to use but unwind, to improve our energies and release those energies that are … the least good in order to be able to then think more rapidly, eloquently and rationally.”*

Thus, increasing physical activity was suggested to minimise the mental health consequences of gaming, performance, and competition by being a tool to “clear up their mind”, reduce mental health symptoms, and improve mental capacity (e.g. focus) and overall energy. Since esports was reported as a "mentally demanding activity", performance is believed to be optimised when players regularly do physical activity.

*José: “I note that when I train [as do exercise], my performance in the game is always better and when I don’t train, some days, I feel that … well, sometimes I have a bit lower level of performance [.] but I really feel this connection and I think that’s why I also believe it’s really important to do sport.”*

Regarding physical health, physical activity was argued to avoid and minimise musculoskeletal problems by counteracting the consequences of being in inadequate postures, rigid positions, and doing repetitive movements.

*Carlos: “Health prevention, I mean…a believe that it’s a important point, besides physical activity.”*

Also, players highlighted that physical activity is important as an alternative to rest from playing and to balance the health risk they face due to high sitting time related to playing esports. This is illustrated by Júlio when referring “*It’s more difficult to be fit. […] we spend a lot of time sat downplaying, I think this clearly affects your physical health.”.*

#### Enhancing Facilities and Equipment

*David: “Investment even in equipment that we know is not going to greatly wear out our … our vision or a good chair. […] The monitors should be high technology monitors […] that in their technology have the means of not being so aggressive on the sight.”*

More appropriate chairs, controllers, and monitors with more advanced technology, developed to minimise risks on both vision and posture, are preferred to prevent physical health problems.

Specifically, to prevent visual problems, different participants added that players should respect the distance between the chair and the monitor.

#### Improving Health Support

*David: “In the preventive sense, even before there are any problems, knowing that they [esports players] are at a higher level and that there is some risk, already preparing themselves and being aware and having support for those earlier signs […] safeguarding against future problems if there is intervention new, immediately, to be able to offset the dangers that may come from these areas.”*

Having access to clinical support, comprising of mental health professionals (e.g. psychologist), medical doctors (e.g. ophthalmologist, team doctor), nutritionists, exercise professionals (e.g. personal trainer), and physical technicians (e.g. physiotherapist), were explained to help prevent or mitigate health consequences of esports practice.

*David: “Making recourse to a specialist […], somebody who is able to accompany us and so if we are not able to understand something that is our problem, or the capacity to perceive what is taking place there, then, it’s always about recourse to a specialist on the subject able to help us.”*

All participants expressed the need for esports teams to have access to a mental health professional regularly to prevent and recognise mental health problems or to help players manage them.

*Joaquim: “I think that psychological support is very important […] I think it’s so important having a person only focused on this, on everything …. everything that the game requires so that, perhaps, we have … for what the life demands … and then everything mixed”*

Moreover, since esports have been reported to demand high mental activity, it was added that psychologists could provide players with strategies to properly cope with the strains of esports competitions to improve performance. In fact, during the interviews, it was consensual regarding the crucial role of a mental health professional (e.g. psychologists, doctors) for prevention, treatment, and performance improvement, being proposed that they could act during daily practice and competitions while working with other support staff to help players.

*César: “At the psychological level, I think it makes sense to have somebody accompanying the players more frequently, that might be able to have more impact […] in competitive environments, having somebody there able to check the mental health of players and be able to better understanding … some of the indications of mental health may bring added advantages.”*

Still, given the lack of structural mental support within esports teams (i.e. only one player stated to have access to certified mental health support within the esports team) or access to qualified personnel, esports players explained that they usually rely on their families, friends, coaches, and team players for mental support during and after competitions and during periods of increased stress related to esports.

*Joaquim: “We end up having our coach who give us, perhaps, this mental support, who also kind of has this function as well and more than at the tactical and technical level.”*

This has been reiterated by coaches, noting their role in supporting esports players during training and, especially, during competition. Moreover, coaches and staff members exemplified their role to foment team cohesion.

Indeed, it was noted that esports is important for socialisation and social life, this being another positive health impact of esports participation to support players’ mental health.

*Joel: “The advantage of esports means you end up having, if you have a team, you always have somebody. So, you’re in this but you’re not in this alone. […] And we do end up creating a family of some type.”*

Besides, mental health support access to a nutritionist in an esports team could be a tool to educate players on better nutritional habits on what to eat or avoid improving performance and well-being while training and competing (i.e. food choices that could enhance mental capacity).

*José: “Now, thinking about the nutritionist… what we should and should not eat, but also the calories that we are consuming, what is it that has more and what has less, what are the options that might be able to help us, for example, in levels of concentration…”*

Most players and staff acknowledged that elite esports players could benefit from having access within their clubs to a medical doctor that evaluated physical problems and a physiotherapist or osteopath to prevent or treat musculoskeletal pain and injuries.

*Júlio: “Perhaps, perhaps it’s that… so if I begin getting pains in my fingers and hands. having a doctor who understands this. who makes x-rays or anything else that may help … . for the pains to go away.”*

To prevent any major health problems, it was proposed by different participants that esports players should be given access to a health screening with a general clinical history and cardiovascular, musculoskeletal, and ophthalmologic health, like the sport’s pre-participation examination.

*Duarte: “having this support and already foreseeing the existence of such situations. people know that there are potential dangers, potential injuries that can come from the exhaustive utilisation involved in these competitions, of practising and playing this sport, perhaps then it would be possible in many cases to delay or prevent something that may end up happening as a direct consequence of this sport.”*

Finally, supporting scientific research related to the health consequences of esports is needed so more efficient strategies can be employed, this being one of the topics highlighted by staff members (i.e. David, César) and one player (i.e. José).

*David: “Sharing, almost constantly sharing these facts so that we are able to gather and increase knowledge about the sport, about what it needs. Connecting this to the particular performance of the clubs, of the players and increasingly involving more specialists, in diverse areas so that they can help in applying more healthcare to virtual football.”*

One suggestion was to learn from what has been studied and found in workers facing similar occupational constraints (e.g. persons in technical or administrative support occupations) and replicate it to improve health prevention and care for esports players, thus improving how health support is given.

*César: “It’s trying to seek a parallelism for something that is very similar or another very similar activity and I think that’s the starting point.”*

Likewise, the findings should be widely shared, so those involved with esports can be aware of the health risks of esports, how health can impact esports performance, and what they might need to improve both health and performance.

## Discussion

Health at its fullest is the highest attainable standard for every human [14]. Some participants also noted that being healthy also means being able to adapt and to self-manage, as stated in recent definition propositions [15, 16]. In the present study, esports participation was discussed to affect esports players' mental and physical health. Overall, participants in the current study recognised that, generally, esports players benefit from esports participation as this activity might improve esports players’ cognitive ability and facilitate mental health when players achieve good performances. Yet, esports participation might also negatively affect mental health by predisposing players to symptoms of common health problems related to esports competition and decreased performance, and affect physical health, as a consequence of using esports equipment for long periods or in an incorrect way and the detrimental effects of spending too much time sitting. To counteract those negative impacts, the participants explained different strategies of health prevention that could be used. Furthermore, some strategies were suggested to play an important role in various forms of prevention (e.g. physical activity).

### The Esports Players’ Health

The importance placed on factors affecting esports players’ health has been recently acknowledged, mostly by qualitative research. Workers and athletes from traditional sports have also discussed physical and mental health problems and share similarities with esports players. For instance, the European Agency for Safety and Health at Work [55] recognised that physical and mental health problems are among the most important occupational safety and health problems in Europe, being frequently connected [55]. Yet, no inclusion and clarification of the impact of esports as a new profession has been made for such entities. Indeed, also for athletes from traditional sports, it has been stated that mental health is connected with physical health (i.e. increased risk of physical injuries and delayed recovery when mental health symptoms and disorders are presented [56]). Hence, even with scarce literature in the esports context, the different factors on esports players' physical and, mainly, mental health have been discussed to impact the overall health [30, 57–59].

### Mental Health

Recent studies on esports found similar conclusions about the mental health impact of gaming, competition, and performance [30, 59]. As explained in the background section, gaming has been previously described to be a factor positively affecting esports players' health, with effects on cognition, improved abilities and skills in a different range of domains (e.g. attention, visuo-spatial, memory, strategy, etc.) that would help players in esports participation and for common daily activities [8, 30]. These positive impacts were widely discussed during the interviews.

Still, as seen in traditional sports [60–62], factors related to esports competitions and performance have also been reported to contribute to mental health symptoms in esports players [27] and acknowledged during the interviews. Additionally, symptoms were found to impair performance, which may also exacerbate symptoms of mental health problems, with impact on overall health [17, 23]. In the present study, the competition itself and the dependence on performance were the factors most affecting players' mental health rather than team-related issues or communication problems previously described and perceived in other esports contexts [18, 24].

Furthermore, as studied for traditional sports, the risk of mental health symptoms in elite athletes is related to physical health problems, decreased performance, or maladaptive perfectionism [56], so an empirical connection between esports players and athletes from traditional sports could be made.

### Physical Health

The most identified themes regarding physical problems were visual and musculoskeletal problems, in line with previous studies [28, 63, 64]. Besides, players acknowledged the known impact of sedentarism on health. Indeed, visual problems related to dark environments with artificial lights, intense light from monitors, or inappropriate distance between players and monitors, have been described [65] and might be responsible for symptoms related to digital eye strain [64, 66, 67]. Furthermore, those environments might lead to other adverse health outcomes by affecting circadian biology, neurobehavioral processes, and health, including disrupted sleep, obesity/diabetes, depression, heart disease, cancer or impaired immune system [68].

As for musculoskeletal problems reported, as also noted with increased esports practice and longer careers, it is expected that problems such as overuse or overtraining injuries might arise [19–21, 63], impacting their performance. Additionally, knowing that esports practice is a sedentary activity, as broadly studied, could predispose players to increase all-cause and cardiovascular mortality risk [69]. Moreover, sedentary time might increase physical problems connected with long hours of competition, poor posture, and screen time. Indeed, posture and working in awkward positions, repetitive work or environmental conditions, as in esports environments, are known physical risk factors related to musculoskeletal problems and injuries in the back, upper limbs, and lower limbs [55]. Fortunately, the participants noted that they preferred having an active lifestyle when not in esports-related activities. This aligned with recent reports in esports and gaming players that showed no adverse associations between video game play time and physical activity [70].

### Strategies to Improve Health in Esports Players

Different strategies to deal with factors affecting esports players' health were suggested during the interviews. Contrary to anecdotal beliefs and previous reports, elite esports players do not need to spend most of their day playing to excel in their practice [57, 71]. Variations on how to train, more efficient and specific scenario practices or training with more skilled or challenging opponents could improve esports training while reducing excessive esports practice [34]. Also, to improve performance, players may need to increase gaming breaks with other activities and reduce esports training duration [22, 58], strategies that were referred to during the interviews. Self-regulated breaks during long play sessions and tournaments, such as simple walks, small breaks with other activities, or long-term removal from esports, have also been suggested in other contexts and esports modalities [24, 72], especially as adaptive coping strategies to help avoid stressors present in esports competitions (e.g. technical issues and antisocial behaviour of other players) [73]. Additionally, those strategies could help prevent mental (e.g. mental fatigue) and physical health (e.g. back pain) problems mentioned to be present in esports players.

Additionally, as explained, esports players should improve their lifestyle habits to promote and prevent health problems. Players recognised that physical activity, nutrition, sleep, and fitness could benefit esports performance [23, 34, 70]. For example, based on studies on dietary patterns for cognition and mental health, healthy diets could give players the extra edge to enhance esports performance and mental health [74–76]. Conversely, knowing that unhealthy eating habits have been associated with cognitive decline and mental health problems, such as depression [74], nutritional literacy may help prevent such problems in a population relying on mental capacity and health. The role of physical activity, including exercise or regular sports practice (i.e. football), was the most stated strategy to improve esports players' health. The positive impact of physical activity was recognised in all factors affecting esports players' health by improving lifestyle habits, enhancing and maintaining physical fitness and performance, optimising esports training, or being an alternative activity to esports practice or refraining from esports. As reflected during the interviews, physical activity might enhance cognitive abilities, like learning, memory, attentional, and executive processes [76, 77]. Likewise, it could minimise common mental health symptoms (e.g. depression, anxiety) and improve psychological well-being and quality of life by improving the feeling of control, self-efficacy, or self-esteem [77], which were frequently associated with mental health during the interviews. Indeed, physical activity is known to be beneficial in people with symptoms of mental health problems, such as distress, anxiety, and depression, especially in healthy individuals, like our participants, showing medium effects when compared with the care normally given [78]. Other general health benefits associated with increased physical activity (e.g. improved all-cause mortality and chronic diseases like hypertension, specific cancers, or type-2 diabetes) should be considered [79]. Likewise, replacing sedentary time with physical activity would provide players additional health benefits by reducing the detrimental effects of high levels of sedentary behaviour and screen time on health (e.g. musculoskeletal complaints [79, 80]. Therefore, comprehensive physical training interventions may help optimise esports skills and maximise performance and health [1, 81].

To avoid other physical health problems, esports players should rely on up-to-date technology, like high technological screen devices and more ergonomic consoles and chairs. Learning from strategies proposed for work-related health problems, possible interventions that should be taken within esports teams could include equipment adaption to esports framework (e.g. ergonomic redesign), and physical activity promotion, besides adjustment of training schedules [82]. Likewise, attention to logistics and events issues, like giving time for players to adapt to the competition setting and to use different computers, peripherals, or monitors, and avoid recurrent changes in the calendar, could have a positive impact on reducing mental stress faced during competitions, besides its impact on physical health [22].

Even acknowledging the need for health prevention and on-time care, a structured esports medical team is not a common reality for most elite esports players [57], including those participating in the current study. Still, all participants mentioned that esports players would benefit from such support. Having mental health support was essential to the esports players' health. Likewise, validated (for esports players) screening tools, like the Sports Mental Health Assessment Tool 1 (SMHAT-1) or the Sports Mental Health Recognition Tool 1 (SMHRT-1), recently proposed by the International Olympic Committee for elite athletes, would have a valuable role for early diagnosis and prevention of mental health problems in esports players [83]. Moreover, since physical problems affect performance and general health, monitoring overuse and overtraining injuries while understanding the risks of physical conditions on health problems development is also needed [12]. Thus, the most suggested support staff in the present study was a medical team comprising psychologists, medical doctors, personal trainers, and physiotherapists or osteopaths. This is similar to the support teams in traditional sports. It aligns with a previously integrated health management model proposed by DiFrancisco-Donoghue et al. [84]. Moreover, education on symptoms and consequences might be a strategy to avoid health problems in esports players and one of the main roles of healthcare professionals working in esports teams. Esports coaches are in a critical position to improve players' literacy about mental health problems, recognition, and treatment, giving them support to seek help when needed [60]. Indeed, close friends and family members would be other potential sources of mental support, particularly when players feel affected by decreased performance and competition tolls. Particularly, esports coaches could minimise the negative impact of competition on elite players by giving players mental support, setting team standards, and developing team interpersonal communication [23, 24]. The coaches interviewed in the present study (and the members of the esports department that had a strong connection with coaches and players of the national team) are aware of their role for mental health support and awareness. As also observed, esports could improve socialisation and social life [9] for factors such as support from esports partners, commitment to team players and team-building activities that have been recognised as beneficial for mental health [57, 85]. Since those activities were mentioned to be part of the common activities on the training camps of the national esports team, team issues within the national team were not mentioned to impact mental health. Instead, the positive effect of esports teams to promote socialisation and social support within esports players and staff members was discussed.

### Practical Applications

Following the arising themes linked to esports health, interventions targeting esports players' mental and physical health (e.g. including primary and secondary stakeholders on health policies and support, improving resources, increasing awareness to esports players' health, learning from what has been done in similar contexts) are needed to improve and protect esports players' health.

First, since many of the current strategies are self-organised, stakeholders engaged with esports (e.g. coaches, support staff, managers, and sponsors) have a key role in promoting the health literacy of esports players, ultimately facilitating esports players' self-efficacy [70]. Improving resources, such as adequate equipment and access to proper healthcare resources, should be a focus for future interventions [55]. As such, the present study highlights that both players and staff members are already aware of the impact of esports participation on health, and mostly share the same beliefs on how to employ the positive effects of esports participation and what could be strategies to prevent health-related problems related to esports participation.

Finally, research integrating esports players is needed to promote a more widespread promotion of physical and mental health, and to substantiate the perceived constraints of esports practice for the esports players' health (e.g. differences in performance due to physical problems, overuse injuries, burnout, sleep problems, coping mechanisms for competition and performance anxiety [27, 72]). Our findings highlight that exercise and health professionals (e.g. sports physicians, psychologists, nutritionists) have an important role in health literacy promotion and support because of the similarities between the health consequences of elite esports practice and sports. Additionally, health literacy and information regarding the potentially negative effect of esports participation could prompt on-time care at first sight of physical problems [28, 82].

### Methodological Considerations

As previously mentioned, the last two interviewed participants did not add any new information about the esports players' health (e.g. they also discussed the two main topics: mental and physical health), thus reaching theme saturation. Although we developed questions, including the follow-up questions, to encourage participants to feel comfortable in the interview, some participants frequently found it challenging to share their experiences which appeared to contribute to the interview duration. We acknowledge that because this is the first qualitative study conducted by AMP, the questioning adopted could be improved. Moreover, the response-driven questions during the interviews could be biased by AMP’s clinical background and her mindset focussed on a health prevention approach.

Additionally, during thematic analysis, a distinction between player and staff member responses was not performed. Contrary to our initial beliefs when designing this study, the participants discussed themes regardless of whether players of staff members were comparable, suggesting a consensual perception of the health factors of esports participation and preventive strategies. This could be related with staff members’ experience with gaming and esports, besides their actual role and specific tasks [86]. Still, even with compatible perceptions, the way they discussed and understood the esports players’ health and strategies proposed to support health might have enriched our study by adding different explanations within the same theme [86, 87].

Lastly, since we only included Portuguese participants associated with eFootball participation, the results discussed here might differ in other esports modalities or national contexts [88, 89], as different esports vary regarding the demands and the characteristics needed to excel [9]. The factors affecting esports players’ health could be related to how health is perceived nationally and the current development of eFootball in Portugal. Still, as a reflexive exercise on the findings and the cited health impact of esports participation, we hope that the results found can be transferable for other esports groups (e.g. national federations and clubs), promoting constructive and action-orientated conversations within esports and traditional sports organisation leaders, coaches and sports and exercise professionals working with esports players [90].

## Conclusion

Elite and world-class eFootball players and staff members considered that specific esports factors (e.g. competitions, logistics) impact players' mental and physical health. Esports players should optimise their lifestyle habits and training, including logistics while relying on social and clinical support to counteract those factors. Also, it is up to esports teams and those belonging to the whole esports ecosystem (e.g. industry and game developers, esports teams, players and followers, and competition organisers) [38] to be aware of the health impact of esports participation, and the strategies proposed here, so they are properly able to offer adequate logistics for their players and to optimise the clinical support given within esports teams, with emphasis on health literacy, health prevention, and support. Given the exponential growth of esports and the relative impact on players, followers and stakeholders, working on factors affecting (positively and negatively) mental and physical health, and defining strategic plans to improve and manage esports players' health will be important for esports teams and all the community involved in this contemporary phenomenon.

## Supplementary Information

**Additional file 1.** **Table 1.** Interview metrics.

**Additional file 2.** Consolidated criteria for reporting qualitative studies (COREQ).

**Additional file 3.** **Supplementary figure 1:** Factors and strategies used to improve esports players' health.

## Data Availability

The datasets generated and/or analysed during the current study are not publicly available due to their containing information that could compromise the privacy of research participants but are available from the corresponding author on reasonable request.

## Abbreviations

eFootball: Electronic football
FPF: Portuguese Football Federation
IOC: International Olympic Committee
WHO: World Health Organisation
