# Supplementary material for: Perspectives of eFootball Players and Staff Members Regarding the Effects of Esports on Health: A Qualitative Study
Source: Sports Med Open. 2023 Jul 26;9:62. doi: 10.1186/s40798-023-00617-0 (PMC10371963; doi:10.1186/s40798-023-00617-0)
Supplement: Supplementary file 1 — Additional file 1. Table 1. Interview metrics. [file 40798_2023_617_MOESM1_ESM.docx]

PERSPECTIVES OF EFOOTBALL PLAYERS AND STAFF MEMBERS REGARDING THE EFFECTS OF ESPORTS ON HEALTH – A QUALITATIVE STUDY

Ana Monteiro Pereira^*1,2^; Caroline Bolling^3^, Phil Birch^4^, Pedro Figueiredo^5,6^, Evert Verhagen^3^, João Brito^1^

^1^Portugal Football School, Portuguese Football Federation, Oeiras, Portugal

^2^Research Center in Sports Sciences, Health, Sciences and Human Development, CIDESD, University of Maia, ISMAI, Maia, Portugal

^3^Amsterdam Collaboration on Health & Safety in Sports, Department of Public and Occupational Health, Amsterdam Movement Sciences, Amsterdam UMC, Vrije Universiteit Amsterdam, Amsterdam, The Netherlands

^4^Institute of Sport, Nursing and Allied Health, University of Chichester, Chichester PO19 6PE, United Kingdom

^5^Physical Education Department, College of Education, United Arab Emirates University, Al Ain, Abu Dhabi, United Arab Emirates

^6^Research Center in Sports Sciences, Health, Sciences and Human Development, CIDESD, Vila Real, Portugal

*Corresponding author: ana.pereira@fpf.pt

# Supplementary material

**Interview metrics**

Table 1. Interview metrics

| Code | Date | Interview duration | Transcript words | Transcript paragraphs | Transcript lines |
| --- | --- | --- | --- | --- | --- |
| Carlos | 07/01/2022 | 00:48:00 | 6451 | 131 | 444 |
| Jaime | 10/01/2022 | 00:28:32 | 4153 | 40 | 343 |
| César | 14/01/2022 | 00:32:20 | 4170 | 124 | 335 |
| Joaquim | 17/01/2022 | 00:28:02 | 3747 | 78 | 259 |
| José | 31/01/2022 | 00:32:18 | 5087 | 86 | 351 |
| Júlio | 01/02/2022 | 00:30:10 | 4907 | 96 | 319 |
| Duarte | 15/02/2022 | 00:55:20 | 7710 | 89 | 496 |
| David | 23/02/2022 | 00:49:00 | 7641 | 81 | 502 |
| Jonas | 07/03/2022 | 00:38:17 | 5147 | 123 | 369 |
| Joel | 12/04/2022 | 00:32:50 | 5129 | 185 | 419 |

Note: Pseudonyms were randomly selected using typical Portuguese names (aligning to the participants' gender) and replaced participant names to assure anonymity. Chosen names consider the initial letter of each name according to participants' activity, (i.e., J=Player, C=Coach, D=esports Department).
