# Supplementary material for: Perspectives of eFootball Players and Staff Members Regarding the Effects of Esports on Health: A Qualitative Study
Source: Sports Med Open. 2023 Jul 26;9:62. doi: 10.1186/s40798-023-00617-0 (PMC10371963; doi:10.1186/s40798-023-00617-0)
Supplement: Supplementary file 2 — Additional file 2. Consolidated criteria for reporting qualitative studies (COREQ). [file 40798_2023_617_MOESM2_ESM.docx]

SPORTS MEDICINE OPEN

PERSPECTIVES OF EFOOTBALL PLAYERS AND STAFF MEMBERS REGARDING THE EFFECTS OF ESPORTS ON HEALTH – A QUALITATIVE STUDY

Ana Monteiro Pereira^*1,2^; Caroline Bolling^3^, Phil Birch^4^, Pedro Figueiredo^5,6^, Evert Verhagen^3^, João Brito^1^

^1^Portugal Football School, Portuguese Football Federation, Oeiras, Portugal

^2^Research Center in Sports Sciences, Health, Sciences and Human Development, CIDESD, University of Maia, ISMAI, Maia, Portugal

^3^Amsterdam Collaboration on Health & Safety in Sports, Department of Public and Occupational Health, Amsterdam Movement Sciences, Amsterdam UMC, Vrije Universiteit Amsterdam, Amsterdam, The Netherlands

^4^Institute of Sport, Nursing and Allied Health, University of Chichester, Chichester PO19 6PE, United Kingdom

^5^Physical Education Department, College of Education, United Arab Emirates University, Al Ain, Abu Dhabi, United Arab Emirates

^6^Research Center in Sports Sciences, Health, Sciences and Human Development, CIDESD, Vila Real, Portugal

*Corresponding author: ana.pereira@fpf.pt

# Supplementary material

**Consolidated criteria for reporting qualitative studies (COREQ)**

Developed from: Tong A, Sainsbury P, Craig J. Consolidated criteria for reporting qualitative research

(COREQ): a 32-item checklist for interviews and focus groups. International Journal for Quality in Health Care. 2007. Volume 19, Number 6: pp. 349 – 357

| No. Item | Guide questions/description | Explanation | Reported on # |
| --- | --- | --- | --- |
| **Domain 1: Research team** | | | |
| Personal characteristics | | | |
| 1. Interviewer | Which author/s conducted the interview or focus group? | AMP conducted all individual semi-structured interviews | Page 6 under section "2.4. Interview guide" |
| 2. Credentials | What were the researcher's credentials? E.g. PhD, MD | AMP, MD, MSc; CB, PhD; PB, PhD; PF, PhD; JB, PhD; EV, PhD | Page 26, under section "5.6. Authors' information" |
| 3. Occupation | What was their occupation at the time of the study? | AMP, PhD candidate, medical doctor; CB Sports physiotherapist and researcher; PB, professor and researcher; PF, researcher; EV, professor; JB, exercise physiologist and researcher. | Page 26, under section "5.6. Authors' information" |
| 4. Gender | Was the researcher male or female? | The interviewer is female, CB is female, and the other authors are male. | Page 26, under section "5.6. Authors' information" |
| 5. Experience and training | What experience or training did the researcher have? | AMP is a PhD candidate and a family physician with a master's in sports medicine; AMP has been studying health-related parameters in esports players and has previously performed a pre-participation health evaluation of the National eFootball team in 2019. CB is a PhD and physiotherapist, experienced with qualitative analysis and research on perceptions of athletes and staff regarding injury and its prevention. PB is a PhD researcher and has previously investigated stress, and mental ill health in esports players, primarily using a qualitative approach. EV is a PhD sport scientist and epidemiologist, experienced in conducting qualitative research, and has limited experience with esports. PF and JB are PhD exercise physiologists and researchers with sports sciences, exercise physiology, and training expertise. | Page 26, under section "5.6. Authors' information" |
| Relationship with participants | | | |
| 6. Relationship established | Was a relationship established prior to study commencement? | No. |  |
| 7. Participant knowledge of the interviewer | What did the participants know about the researcher? E.g. personal goals, reasons for doing the research | Participants were informed of the background and aims of the study before the interview in the invitation to participate. Participants were briefed on the purpose of the study and understood it. Ethical had granted, and participants reviewed the participant information documentation before giving their written informed consent to be involved. They were explained the medical background of AMP and provenance for the study. | Page 7, under sections" 2.2 Procedures" and "2.3. Data collection" |
| 8. Interviewer characteristics | What characteristics were reported about the interviewer/facilitator? e.g. Bias, assumptions, reasons and interests in the research topic | At the beginning of the interviews, AMP explained that she has been studying health-related parameters in esports players as a part of her PhD. AMP had previously performed a pre-participation exam on the FPF eFootball national team. She is a family doctor working in Portugal and a team doctor in the Portuguese Football Federation. |  |
| Domain 2: Study design | | | |
| Theoretical framework | | | |
| 9. Methodological orientation and Theory | What methodological orientation was stated to underpin the study? e.g. grounded theory, discourse analysis, ethnography, phenomenology, content analysis | A critical realist perspective approach was used. | Page 6, Under section "2.1 Ontological and epistemological assumptions" |
| Participant selection | | | |
| 10. Sampling | How were participants selected? e.g. purposive, convenience, consecutive, snowball | A purposive sampling was used. | Page 6, under section "2.3. Procedures" |
| 11. Method of approach | How were participants approached? e.g. face-to-face, telephone, mail, email | The FPF eFootball department provided a list of potential participants for the study. Those who expressed interest in participating gave their contact details to directly arrange the interview with AMP by e-mail. Additionally, participants were asked to provide the contact of other further potential participants. | Page 6, under section "2.3. Procedures" |
| 12. Sample size | How many participants were in the study? | In total 10 participants. | Page 6-7, under section "2.3. Data collection" |
| 13. Non- participation | How many people refused to participate or dropped out? Reasons? | Eight potential participants (players) did not respond after three contact attempts. | Page 6-7, under section "2.3. Data collection" |
| Setting | | | |
| 14. Setting of data collection | Where was the data collected? e.g. home, clinic, workplace | All data was collected using Microsoft Teams. | Page 7, Under section "2.4. Interview guide" |
| 15. Presence of non-participants | Was anyone else present besides the participants and researchers? | All interviews were conducted without anyone else present. | Page 7, Under section "2.4. Interview guide" |
| 16. Description of sample | What are the important characteristics of the sample? e.g. demographic data, date | They were described under the section "3.1. Sample characterization". | Page 19, under section “3.1. Sample Characterization” |
| Data collection | | | |
| 17. Interview guide | Were questions, prompts, guides provided by the authors? Was it pilot tested? | This is outlined in the Methods section. | Page 7, Under section "2.4. Interview guide" |
| 18. Repeat interviews | Were repeat interviews carried out? If yes, how many? | No. |  |
| 19. Audio/visual recording | Did the research use audio or visual recording to collect the data? | All interviews were video recorded. | Page 8, under section "2.5. Data analysis" |
| 20. Field notes | Were field notes made during and/or after the interview or focus group? | Personal notes were made during data collection. |  |
| 21. Duration | What was the duration of the interviews or focus group? | The mean duration of the interviews was 37±10 minutes (range: 28-55 minutes; see supplementary material). | Page 9, under section "3. Results" and Table 1 in supplementary material |
| 22. Data saturation | Was data saturation discussed? | Data saturation was reached after 10 interviews. | Page 7, under section "2.3. Data collection" |
| 23. Transcripts returned | Were transcripts returned to participants for comment and/or correction? | No. |  |
| Domain 3: Analysis and findings | | | |
| Data analysis | | | |
| 24. Number of data coders | How many data coders coded the data? | AMP independently coded all interviews and then discussed the data with CB to develop themes. JB sense-check the preliminary themes and report. | Page 8, under section "2.5. Data analysis" |
| 25. Description of the coding tree | Did authors provide a description of the coding tree? | No. |  |
| 26. Derivation of themes | Were themes identified in advance or derived from the data? | The themes were data-driven. | Page 8, under section "2.5. Data analysis" |
| 27. Software | What software, if applicable, was used to manage the data? | Data was organized in an Excel file. |  |
| 28. Participant checking | Did participants provide feedback on the findings? | Participants did not provide feedback, but the published paper will be sent to all participants. |  |
| Reporting | | | |
| 29. Quotations presented | Were participant quotations presented to illustrate the themes/findings? Was each quotation identified? e.g. participant number | Yes, within the main text of the "Results" section. |  |
| 30. Data and findings consistent | Was there consistency between the data presented and the findings? | Yes. | It was explained throughout the manuscript. |
| 31. Clarity of major themes | Were major themes clearly presented in the findings? | Yes. The major themes are presented in the sections and subsections under "Results" and Table 1. | Page 9, Table 1. |
| 32. Clarity of minor themes | Is there a description of diverse cases or discussion of minor themes? | Yes. | Page 8, under section "2.5. Data analysis" |
