# Supplementary material for: Perspectives of eFootball Players and Staff Members Regarding the Effects of Esports on Health: A Qualitative Study
Source: Sports Med Open. 2023 Jul 26;9:62. doi: 10.1186/s40798-023-00617-0 (PMC10371963; doi:10.1186/s40798-023-00617-0)
Supplement: Supplementary file 3 — Additional file 3. Supplementary figure 1: Factors and strategies used to improve esports players' health. [file 40798_2023_617_MOESM3_ESM.pptx]

## Slide 1
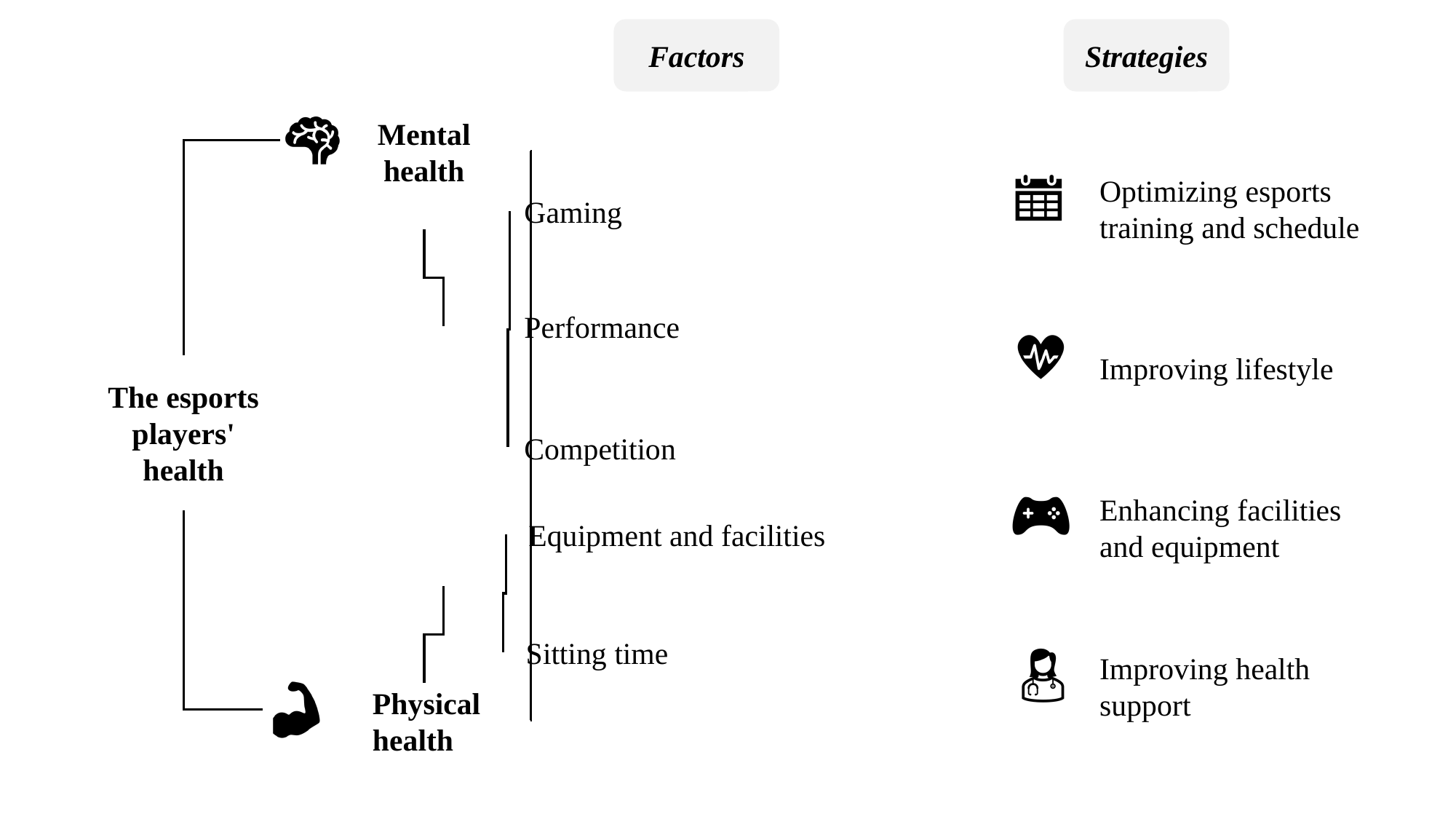

Factors
Strategies
Mental health
Optimizing esports training and schedule
Gaming
Performance
Competition
Improving lifestyle
The esports players' health
Enhancing facilities
and equipment
Equipment and facilities
Sitting time
Improving health support
Physical health
